# Bruton’s tyrosine kinase inhibitor-related cardiotoxicity: The quest for predictive biomarkers and improved risk stratification

**DOI:** 10.18632/oncotarget.28589

**Published:** 2024-06-03

**Authors:** Jai N. Patel, Jai Singh, Nilanjan Ghosh

**Affiliations:** ^1^Department of Cancer Pharmacology and Pharmacogenomics, Atrium Health Levine Cancer Institute, Charlotte, NC 28204, USA; ^2^Atrium Health Wake Forest Baptist Comprehensive Cancer Center, Winston-Salem, NC 27157, USA; ^3^Sanger Heart and Vascular Institute, Atrium Health, Charlotte, NC 28204, USA; ^4^Department of Hematologic Malignancies and Blood Disorders, Atrium Health Levine Cancer Institute, Charlotte, NC 28204, USA

**Keywords:** BTK inhibitor, cardiotoxicity, biomarkers, risk, genetics

## Abstract

Ibrutinib was the first Bruton's tyrosine kinase (BTK) inhibitor approved for the treatment of patients with chronic lymphocytic leukemia (CLL). While producing durable responses and prolonging survival, roughly 20–25% of patients experience dose limiting side effects, mostly consisting of cardiovascular toxicities like severe hypertension and atrial fibrillation. While clinical predictors of BTK inhibitor-related cardiotoxicity have been proposed and may aid in risk stratification, there is no routine risk model used in clinical practice today to identify patients at highest risk. A recent study investigating genetic predictors of ibrutinib-related cardiotoxicity found that single nucleotide polymorphisms in KCNQ1 and GATA4 were significantly associated with cardiotoxic events. If replicated in larger studies, these biomarkers may improve risk stratification in combination with clinical factors. A clinicogenomic risk model may aid in identifying patients at highest risk of developing BTK inhibitor-related cardiotoxicity in which further risk mitigation strategies may be explored.

## INTRODUCTION

### BTK inhibitor-related cardiotoxicity: Scope of the problem

The development and approval of ibrutinib, the first Bruton’s tyrosine kinase (BTK) inhibitor, revolutionized management of B-cell malignancies like chronic lymphocytic leukemia (CLL) [1]. While ibrutinib produces durable responses and randomized controlled clinical trials have demonstrated that it has a generally favorable side-effect profile, real-world data has revealed higher discontinuation rates due to drug-related toxicities (20-25%), particularly cardiovascular side effects (CVSEs), including atrial fibrillation, ventricular arrhythmias, and severe hypertension [2]. A meta-analysis of eight randomized trials demonstrated that treatment with ibrutinib increased the incidence of severe hypertension by 3-fold and atrial fibrillation by more than 4-fold [3]. While CVSEs with second generation BTK inhibitors are less, 2-9% continue to experience arrhythmias, while comparable rates of hypertension between BTK inhibitors suggest a class effect [4]. Notably, development of BTK-inhibitor-related CVSEs are associated with worse long-term CV and overall survival [5].

It has been postulated that CVSEs are attributed to off-target interactions with other kinases, including IL2-inducible T-cell kinase (ITK) and tec protein kinase (TEC), which are heavily expressed on cardiac tissue [6, 7]. The PI3K-Akt pathway, a critical regulator of cardiac protection, is also regulated by BTK and TEC [6]. In addition, it is hypothesized that atrial fibrillation is related to inhibition of Fyn, mitogen-activated protein/extracellular signal regulated protein kinase 5, or C-terminal Src kinase (CSK). Cardiac knockout produced similar effects to those observed with ibrutinib treatment [8].

In the absence of selective prediction tools, optimal management of ibrutinib-related CVSEs involves multidisciplinary collaboration among cardio-oncologists and oncologists to guide shared decision making. However, due to the general lack of robust predictive biomarkers, a standardized risk stratification tool is not utilized in clinical practice – a prime area for research.

### Clinical predictors of cardiotoxicity

In one meta-analysis of 1,505 ibrutinib-treated patients, age (older than 65) and history of atrial fibrillation were associated with the risk of developing ibrutinib-related atrial fibrillation [9]. Another study found that history of atrial fibrillation and Framingham Heart Study AF risk score were significantly associated with the development of atrial fibrillation [10], whereas a similar study found that heart failure and left atrial abnormality on electrocardiogram were independent predictors of atrial fibrillation [11]. As expected, the presence of pre-existing cardiovascular disease (CVD) confers an increased risk of atrial fibrillation. A study by Shanafelt et al. found that older male patients and those with baseline valvular heart disease and hypertension had a higher risk of atrial fibrillation. A predictive model combining these factors stratified patients into four risk groups with 10-year rates of atrial fibrillation ranging from 4% to 33% [12]. A smaller study but with long-term follow-up of 52 months found that pre-existing hypertension, history of atrial fibrillation, and a high Shanafelt risk score were associated with atrial fibrillation incidence after ibrutinib. Baseline echocardiographic evaluation of left atrial dimensions also predicted atrial fibrillation risk [13]. To our knowledge, the risk score proposed by Shanafelt et al. is the only proposed predictive model, which notably only incorporates clinical factors and has not yet been widely adopted in the clinical setting.

### Genetic predictors of cardiotoxicity

Clinical plus genetic risk factors have been studied for various cancer- and treatment-related complications. For example, the ONCOTHROMB score, which considers both clinical and genetic variables, better identified patients at risk for venous thromboembolism and who might benefit from primary thromboprophylaxis [14]. This clinicogenomic score outperformed the clinical only Khorana score. No such score exists for predicting CVSE in patients with cancer.

A recent study identified three single nucleotide polymorphisms associated with ibrutinib-related CVSEs: *GATA4* rs804280, *KCNQ1* rs163182, and *KCNQ1* rs2237895 [15]. A custom next generation sequencing panel was developed to genotype 40 SNPs in *GATA4*, *KCNQ1*, *KCNA5*, *NPPA*, *SCNA5*, and *SGK1*. These genes were identified using Ingenuity Pathway Analysis to generate a network of BTK signaling pathways to search for off target effects related to CVSE. Based on multivariate analyses, a high genetic risk score, defined as the presence of at least two of these genotypes, was associated with 11.5-fold increased odds of CVSEs (*P* = 0.019; 95% confidence interval, 1.79–119.73). Age, race, and weight were the only clinical variables included in the multivariate analysis, none of which were significantly associated with CVSEs. In silico analysis using the GTEx database demonstrated a significant association between rs804280 genotypes and gene expression. Although this study was small (*N* = 50 patients with CLL), it clearly alludes to the possibility that genetics may influence CVSE risk after ibrutinib treatment. These findings require replication in a larger independent cohort, preferably in patients with CLL with prospectively collected toxicity data. A critical question is whether these findings also replicate with newer generation BTK inhibitors, including acalabrutinib and zanubrutinib.

The previously mentioned study used a candidate gene approach based on pathway analysis, thus limiting the potential to validate other known polymorphisms in genes related to hereditary CVD. In fact, it is estimated that there are more than 100 kinds of monogenic CVD, including cardiomyopathy, cardiac ion channel diseases, monogenic inherited hypertension, inherited aortic diseases, pulmonary hypertension, inherited thrombophilia, familial hypercholesterolemia, and others [16]. A previously published comprehensive review describes three classifications in which common gene mutations can influence CVD risk in cancer: inflammation, metabolism, and cell proliferation [17]. Further research is needed to determine the clinical utility of genetic testing for inherited CVD in patients with cancer.

### Current recommendations for screening and managing BTK inhibitor-related cardiotoxicity

An international consensus group of one dozen physicians published recommendations for managing BTK-related cardiotoxicity [18]. The pretreatment workup should include a comprehensive history, medication reconciliation, review of CV risk factors, and CV examination, including electrocardiogram (echocardiogram may be considered in those at high CV risk or with established CV disease). For patients with no CV risk factors, any approved BTK inhibitor may be appropriate. If safety concerns exist (e.g., patient has CV risk factors), one should favor more selective drugs like acalabrutinib or Zanubrutinib (although newer generation BTK inhibitors have now shown improved efficacy over ibrutinib in the relapse/refractory setting and are also approved for front-line CLL). BTK inhibitors are not recommended for patients with history of ventricular arrhythmia, family history of sudden cardiac death, severe uncontrolled hypertension, or severe or uncontrolled congestive heart failure. Importantly, patients with CV risk factors (especially if uncontrolled) should be referred to a cardio-oncologist to ensure CVD is adequately managed before initiating BTK inhibitor treatment. If CVSEs develop, they should be diagnosed quickly and risks versus benefits of holding BTK inhibitor treatment, dose reduction, or switching to an alternative BTK inhibitor should be discussed with a multidisciplinary team including a hematologist and cardio-oncologist. Monitoring frequency for various CVSEs depends on the patient’s risk. For example, if the patient has a history of hypertension, then close monitoring of blood pressure at home biweekly may be appropriate. Otherwise, periodic echocardiograms or other assessments of ejection fraction every six to twelve months may be appropriate for routine monitoring. For patients with emerging CHF, the BTK inhibitor should be stopped immediately, and treatment should be initiated with an angiotensin converting enzyme inhibitor or angiotensin receptor blocker plus beta blocker as tolerated.

The American Heart Association has also published a scientific statement on the prevention and management of arrhythmias and autonomic disorders in cardio-oncology (not specific to BTK inhibitors alone). Specific medication interventions are described based on rhythm and rate control, as well as anticoagulation management based on thromboembolism and bleeding risk, the latter which is known to be increased with BTK inhibitors [19].

A key concept in cardio-oncology is the idea of permissive cardiotoxicity which is a proactive approach to screening and management of cardiac comorbidities and side effects with the goal of how best to safely continue cancer therapy and avoid interruptions [20]. A combined clinical and genetic BTK inhibitor cardiotoxicity risk model outperforming current clinical risk models would be ideal in personalized multidisciplinary care with a focus on high specificity, good sensitivity, and a low misclassification rate. In those individuals deemed high risk and initiated on a BTK inhibitor, high specificity will allow confidence in targeted closer monitoring including serial cardiac rhythm and blood pressure monitoring as well as tailoring management strategies from medical therapies to catheter-based atrial fibrillation ablation as needed. Equally important is avoiding overestimation of risk and preventing withholding of lifesaving BTK inhibitor treatment in patients who are not truly high risk, and future prospective validation studies should include those with a history of CVD including CHF, coronary artery disease, and ventricular arrythmias that are well compensated.

## CONCLUSION

The American Heart Association issued a scientific statement in 2020 on genetic testing for inherited CVDs (not specific to cancer), highlighting the potential value of genetic testing to screen for known inherited CV conditions [21]. Risk stratification for cardiotoxicity in patients with cancer receiving anticancer treatment is critical [22]. While several clinical predictors of cardiotoxicity exist in patients with cancer, there is a paucity of data on the use of genetics for further risk stratification. At least one study has identified candidate SNPs associated with increased CVSE risk with ibrutinib [15], which still requires replication in a larger independent cohort. There is a major need for the development and validation of a clinicogenomic algorithm for CVSE risk stratification and prospective studies evaluating the clinical utility of such an algorithm to test various risk mitigation strategies. This would allow a more personalized approach to selection of anticancer treatments, CV monitoring, implementation of pharmacologic or non-pharmacologic interventions, all in hopes of improving survival in patients with cancer.
